# Moving silicone oil particles in the ventricle: a case report and updated review

**DOI:** 10.1186/s12886-022-02328-8

**Published:** 2022-03-01

**Authors:** Shugang Cao, Hao Zhao, Jian Wang, Jun He, Mingwu Xia, Wen’an Xu

**Affiliations:** Department of Neurology, Hefei Hospital Affiliated to Anhui Medical University, No. 246 Heping Road, Hefei, 230011 China

**Keywords:** Silicone oil, Retinal detachment, Ventricle, Imaging, Case report

## Abstract

**Background:**

The movement of intraventricular silicone oil observed in the supine position is extremely rare. Herein, we describe a patient who presented with dynamically moving silicone oil particles in the ventricle when changing position and provide an updated review of this phenomenon.

**Case presentation:**

We report a case of a 70-year-old woman who presented with intraventricular hyperdensities that were occasionally found on brain computed tomography (CT). Initial nonenhanced brain CT demonstrated nondependent hyperdensities in the bilateral anterior horns of the lateral ventricles, the third ventricle, and the right suprasellar cistern, mimicking an intraventricular hemorrhage. Further brain magnetic resonance imaging (MRI) in the supine position revealed abnormal signals in the bilateral anterior horns of the lateral ventricles, the posterior horn of the right lateral ventricle, the third ventricle, the right suprasellar cistern, and the bilateral eyeballs, with isosignal intensities surrounded by low-signal chemical shift artifacts on T_1_-weighted imaging and variable signals (hypo- or hyperintensity) on T_2_-weighted imaging. The lesion in the anterior horn of the right ventricle largely moved to the posterior horn of the ipsilateral ventricle. The final craniocervical CT angiography showed that the lesion in the posterior horn had moved back to the anterior horn of the right lateral ventricle. These features were consistent with intraventricular silicone oil migration. The final spinal MRI did not demonstrate a migration of silicone oil into the spinal subarachnoid space.

**Discussion and conclusions:**

This case report describes a dynamic process of silicone oil displacement in the supine position and provides a comprehensive imaging presentation. The moving pattern and a characteristic chemical shift artifact on MRI are key to the diagnosis and may help prevent unnecessary examinations or intervention.

## Background

Intraocular injection of silicone oil has been used for many years to treat complex retinal detachment. The known complications of intraocular silicone oil tamponade are associated with the physical and chemical properties and the migration of silicone oil [1]. However, silicone oil migration from the vitreous body to the ventricle along the optic nerve and chiasm is extremely rare. A review of the literature indicates that to date, only 32 cases of silicone oil migration into the ventricular system have been reported, including the present case (see Table 1) [2–32]. In 1983, Ni et al. [33] pathologically confirmed silicone oil migration into the optic nerve for the first time and found that silicone oil vesicles were present in the patient's optic disc and optic nerve. Williams et al. [2] reported the first case of silicone oil migration into the lateral ventricle in 1999. The moving pattern of the silicone particles, especially in the prone position, and a characteristic chemical shift artifact on brain magnetic resonance imaging (MRI) are key to the diagnosis [3, 4, 6, 10–14, 16, 26, 27, 29]. Herein, we describe a patient diagnosed with silicone oil migration into the ventricular system when performing subsequent brain MRI in the supine position and provide an updated review of the literature on intraventricular silicone oil migration.

**Table 1:** Reported patients with intraventricular migration of silicone oil

	Author, year	Age/sex	Indication for endotamponade/eye (silicone oil location)	Endotamponade time	Initial location	Prone imaging	Spontaneous movement/ secondary location	CT (HU)	MRI			Treatment
									T_1_WI	T_2_WI	Chemical shift	
1	Williams et al. [2], 1999	42/M	RD (CMV)/Left	15 months	Left LV (frontal horn)	No	Yes/Bilateral LVs (frontal horn)	No	Hyper	Hypo	Yes	No
2	Dong et al. [3], 2005	62/F	RD (diabetic retinopathy)/Right	8 months	3rd V, 4th V and right LV (frontal horn)	Yes	Yes/Right LV (posterior horn)	Yes (NR)	Hyper	Hypo	Yes	No
3	Eller et al. [4], 2000	42/M	RD (CMV)/Left	6.5 months	Bilateral LVs (frontal horn)	Yes	Yes/Left LV (posterior horn)	No	Hyper	Hyper	Yes	No
4	Yu et al. [5], 2005	47/M	Vitreous hemorrhage (diabetic retinopathy)/Right	15 months	Bilateral LVs (frontal horn)	No	Yes/Left LV (temporal horn)	Yes (90)	Hyper	NR	Yes	No
5	Kuhn et al. [6], 2006	15/F	RD (cystic macular edema)/Left	Nearly 4 years	Bilateral LVs (frontal horn)	Yes	Yes/Bilateral LVs (occipital horn)	No	NR	Hyper	Yes	No
6	Tatewaki et al. [7], 2011	66/F	RD (diabetic retinopathy)/Left	NR	Right LV (frontal horn)	No	No	Yes (80)	Hyper	Hypo	Yes	No
7	Jabbour et al. [8], 2011	72/M	RD (diabetic retinopathy)/Right	15 years	Bilateral LVs (frontal horn)	No	No	Yes (NR)	NR	NR	Yes	No
8	Chen et al. [9], 2011	39/M	Diabetic retinopathy/Left	NR	Bilateral LVs (frontal horn)	No	Yes/Right LV (temporal horn)	Yes (82)	Hyper	Hyper	Yes	No
9	Lee et al. [10], 2011	56/M	RD/Left	NR	Left LV (frontal horn)	Yes	Yes/Left LV (occipital horn)	Yes (NR)	NR	NR	Yes	No
10	Hruby et al. [11], 2013	51/M	Diabetic retinopathy/NR	5 years	Bilateral LVs (frontal horn)	Yes	Yes/Bilateral LVs (occipital horn)	Yes (NR)	NR	Hyper	Yes	**Yes**
11	Campbell et al. [12], 2013	51/M	NR/Right	NR	Left LV (frontal horn)	Yes	Yes/Left LV (occipital horn)	Yes (89)	Hyper	Hypo	Yes	No
12	Chang et al. [13], 2013	58/F	RD (diabetic retinopathy)/Left	10 years	4th V and right LV (frontal horn)	Yes	Yes/Left LV (occipital horn)	Yes (86)	Hyper	Hypo	Yes	No
13	Cosgrove et al. [14], 2013	74/F	Diabetic retinopathy/Left	20 years	Right LV (frontal horn)	Yes	Yes/Right LV (occipital horn)	Yes (NR)	Intermediate	Hyper	Yes	No
14	Sato et al. [15], 2014	87/F	RD/Right	NR	Bilateral LVs (frontal horn)	No	No	Yes (NR)	No	No	No	No
15	Chiao et al. [16], 2015	80/F	NR/Left	NR	Bilateral LVs (frontal horn)	Yes	Yes/Bilateral LVs (occipital horn)	Yes (NR)	No	No	No	No
16	Dababneh et al. [17], 2015	73/F	NR/NR	25 years	4th V and left LV (temporal horn)	No	Yes/4th V and right LV (frontal horn)	Yes (NR)	Hyper	Variable	Yes	No
17	Swami et al. [18], 2015	68/M	RD (diabetic retinopathy)/Both	9 ~ 10 years	3rd V	No	Yes/3rd V and bilateral LVs (frontal horn)	Yes (50–60)	No	No	No	No
18	Mathis et al. [19], 2016	82/F	RD/Left	38 months	Bilateral LVs (bilateral frontal horns and left temporal horn)	No	Yes/Bilateral LVs (right frontal horn and left temporal horn)	Yes (NR)	No	No	No	No
19	Boren et al. [20], 2016	82/F	RD (diabetic retinopathy)/Right	9 years	Bilateral LVs (bilateral frontal horns and right temporal horn)	No	No	Yes (75)	No	No	No	No
20	Sarohia et al. [21], 2016	51/F	RD/Left	NR	4th V and right LV (frontal horn)	No	Yes/3rd V, 4th V and right LV (frontal horn)	Yes (100)	Hyper	NR	Yes	No
21	Filippidis et al. [22], 2017	67/F	RD/Left	6 years	Bilateral LVs (frontal horn)	No	No	Yes (NR)	Hyper	Hyper	Yes	No
22	Gnanalingham et al. [23], 2017	84/F	RD/Left	1 year	Bilateral LVs (frontal horn)	No	No	Yes (NR)	No	No	No	No
23	Lin et al. [24], 2018	67/M	RD (diabetic retinopathy)/Left	NR (in 2012)	Bilateral LVs (bilateral frontal horns and left temporal horn)	No	No	Yes (NR)	Hyper	Hypo	Yes	No
24	Mayl et al. [25], 2018	27/M	NR/Left	NR	Left LV (frontal horn)	No	No	Yes (106–139)	Hyper	Hyper	Yes	No
25	Potts et al. [26], 2018	56/F	RD (diabetic retinopathy)/Left	NR (in 2009)	Bilateral LVs (frontal horn)	Yes	Yes/Bilateral LVs (posterior bodies)	Yes (NR)	Hyper	Hyper	Yes	No
26	Carneiro et al. [27], 2019	54/M	RD/Left	NR	Bilateral LVs (frontal horn)	Yes	Yes/Bilateral LVs (occipital horns)	Yes (NR)	Hyper	Hypo	Yes	No
27	Cao et al. [28], 2019	63/M	RD (diabetic retinopathy)/ Right	2.5 years (in 2009)	Bilateral LVs (frontal horn)	No	Yes/Left LV (frontal horn)	Yes (NR)	Hyper	Hyper	Yes	No
28	Cao et al. [29], 2019	77/F	RD (diabetic retinopathy)/ Left	NR	Bilateral LVs (frontal horn)	Yes	Yes/Left LV (occipital horn)	Yes (NR)	No	No	No	No
29	Zhong et al. [30], 2019	49/M	RD (trauma)/ Right	4 years	Bilateral LVs (frontal horn)	No	Yes/Left LV (temporal horn first and frontal horn later)	Yes (NR)	Hyper	Intermediate	Yes	No
30	Shimazaki et al. [31], 2021	62/M	RD (diabetic retinopathy)/ Right	NR	Right LV (temporal horn)	No	Yes/Bilateral LVs (frontal horn)	Yes (NR)	NR	Hyper	Yes	No
31	Mazzeo et al. [32], 2021	67/F	RD (diabetic retinopathy)/ Right	NR (in 2016)	Right LV (frontal horn)	No	No	No	Hyper	Hypo	Yes	Yes
32	This case, 2019	70/F	RD (diabetic retinopathy)/Both	2.5 years	3rd V and bilateral LVs (frontal horn)	Yes	Yes/Bilateral LVs (bilateral frontal horns and right occipital horn)	Yes (70–82)	Hyper	Variable	Yes	No

*Note*: *HU* Hounsfield unit, *RD* Retinal detachment, *CMV* Cytomegaloviral retinitis, *LV* Lateral ventricle, *V* Ventricle, *NR* Not reported

## Case presentation

A 70-year-old female presented with intraventricular hyperdensities that were occasionally found on computed tomography (CT). She experienced complicated retinal detachment in both eyes due to diabetic retinopathy and underwent vitrectomy and bilateral silicone oil tamponade 2.5 years prior. The patient’s intraocular pressure was normal after the operation. However, her vision recovery was poor. Physical examination showed the following: no light perception in the right eye; finger count at 30 cm in the left eye; an oval right pupil with a maximum diameter of approximately 3 mm; a round left pupil with a diameter of 2.5 mm and no light reaction; right eye exotropia with slight limitation of adduction; and normal results for other items of the neurological examination.

**Fig. 1:**
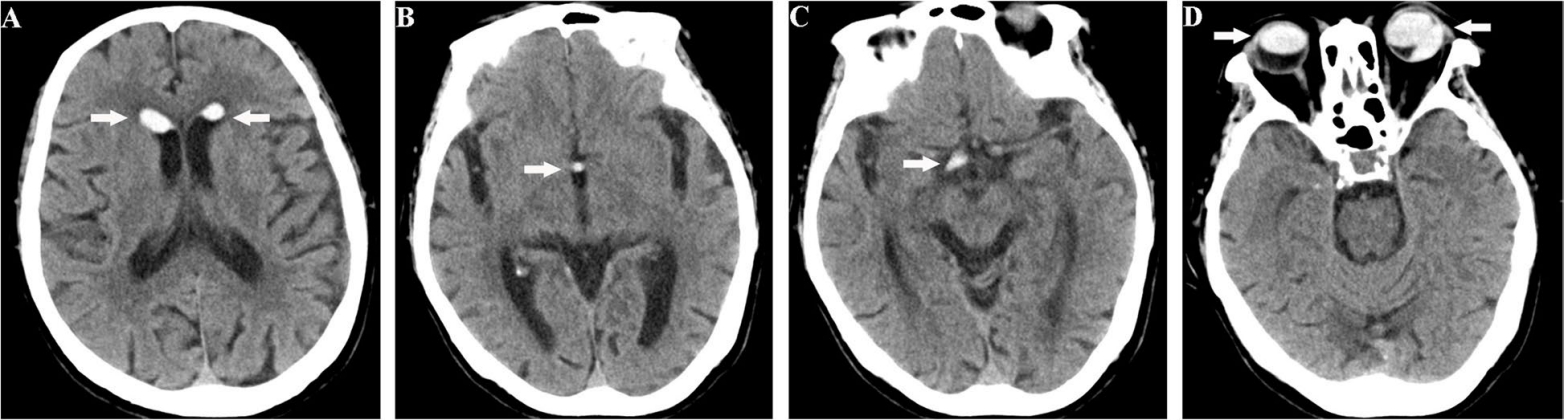
Nonenhanced brain CT demonstrated nondependent hyperdensities in the frontal horns of the bilateral ventricles, in the third ventricle, and the right suprasellar cistern (**A**-**C**, white arrows), as well as in the bilateral eye globes (**D**, white arrows). The CT values of these silicone particles ranged from 70 to 82 HU

**Fig. 2:**
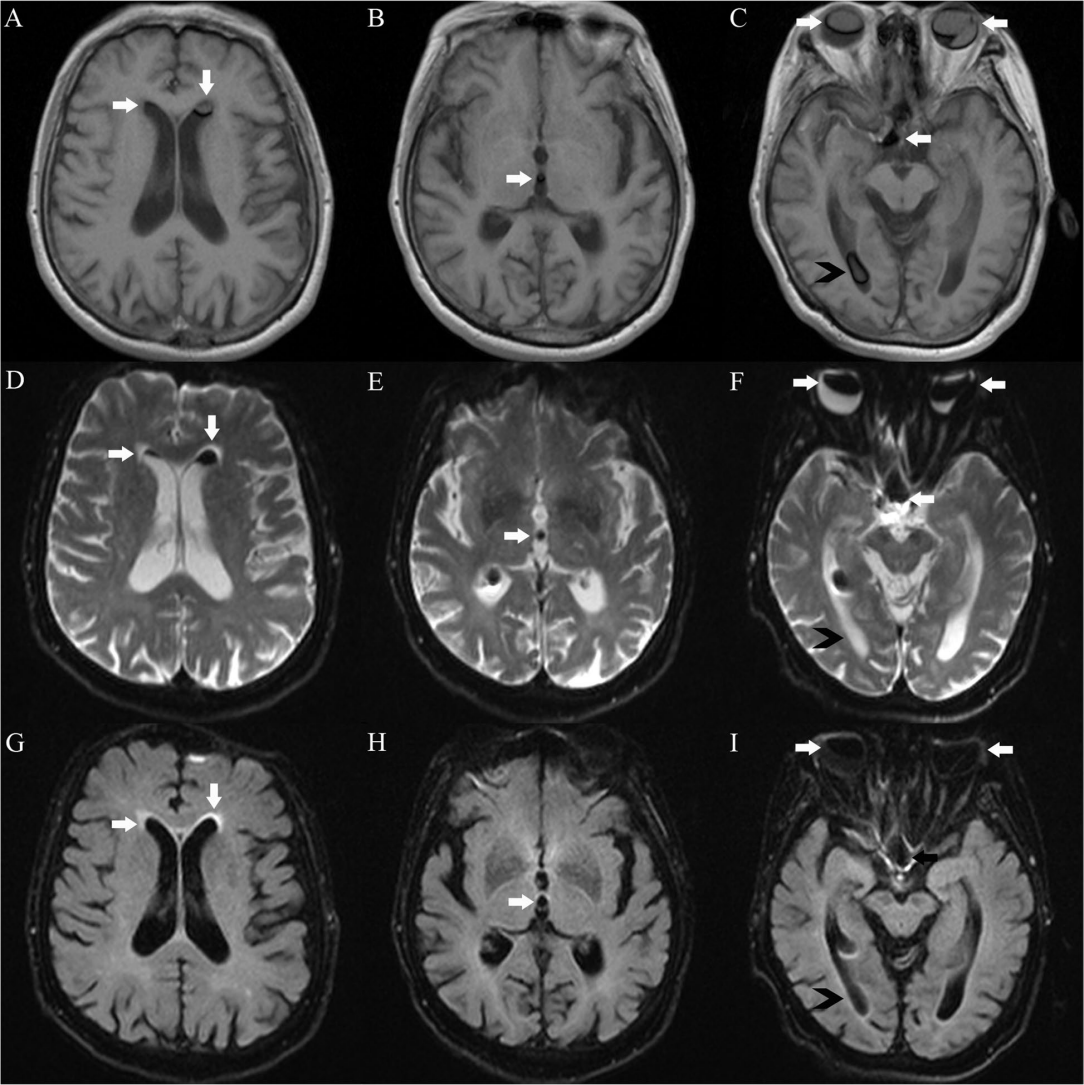
Brain MRI revealed abnormal signals in the bilateral eyeballs, bilateral lateral ventricle anterior horns, right lateral ventricle posterior horn, third ventricle, and right suprasellar cistern, showing hyperintensity (relative to the cerebrospinal fluid), surrounded by low-signal chemical shift artifacts, on T_1_WI (**A**-**C**); variable signals (hypo- or hyperintensity, white arrows and black arrows, respectively) on T_2_WI (**D**-**F**); hypointensity on FLAIR (**G**-**I**). The silicone particles in the anterior horn of the right lateral ventricle on MRI (2A and 2D, white arrows) was smaller than that on CT (1A, white arrow) and mostly shifted to the posterior horn of the right lateral ventricle (2C, 2F, and 2I, black arrows)

**Fig. 3:**
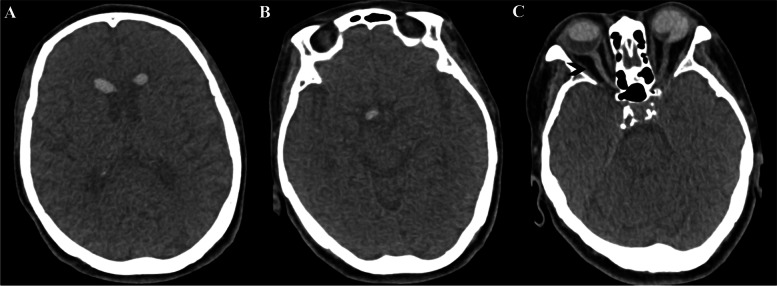
Final craniocervical CTA revealed that the silicone particles in the posterior horn had moved back to the anterior horn of the right lateral ventricle, as shown on nonenhanced brain CT, and demonstrated no enhancement of these silicone particles (**A**-**B**). The right optic nerve had a larger density than the left, which was close to intraocular silicone oil (**C**, black arrow)

**Fig. 4:**
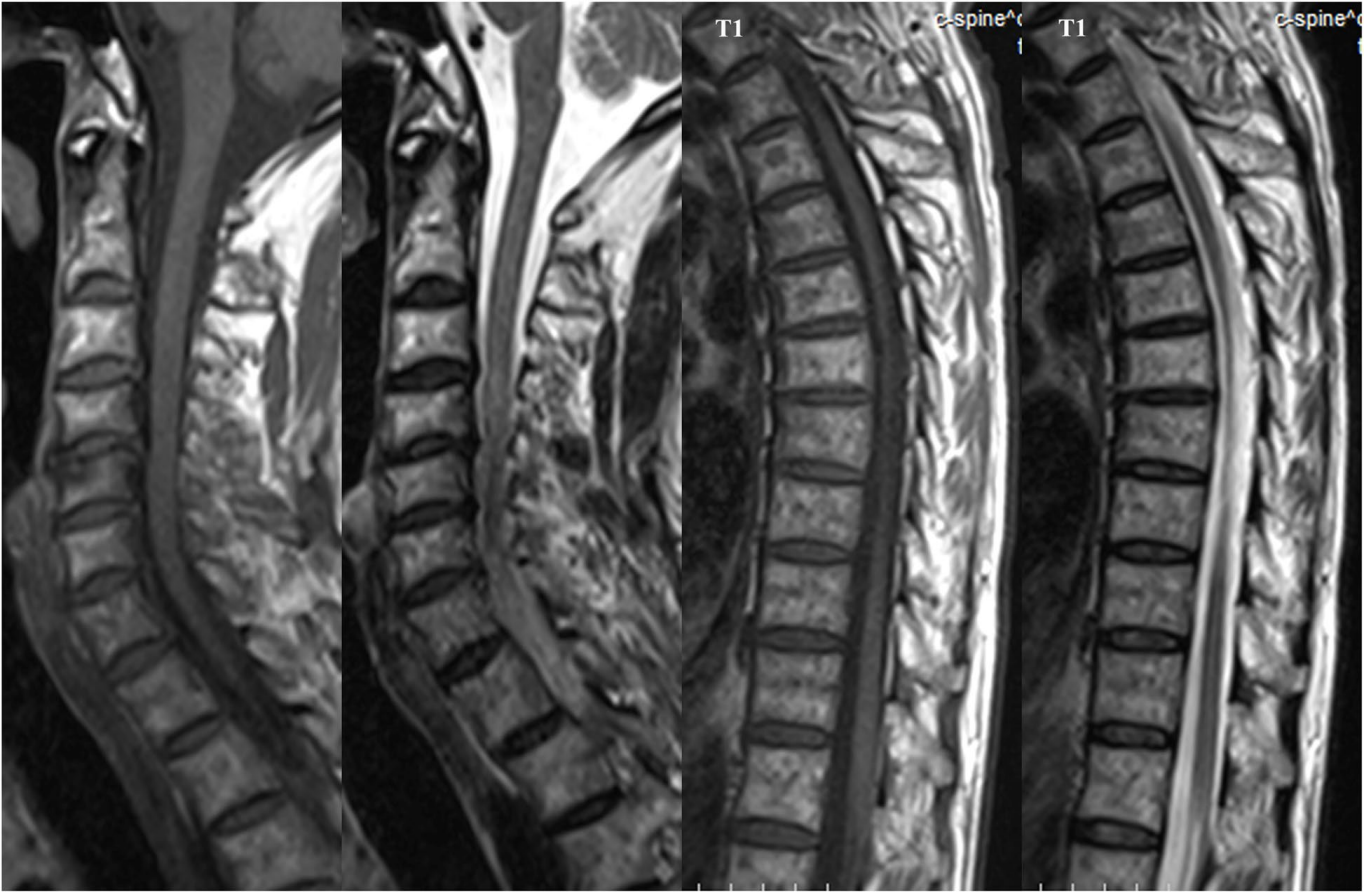
Spinal MRI did not demonstrate silicone oil migration into the subarachnoid space of the spinal cord

Initial brain CT showed nondependent hyperdensities in the bilateral anterior horns of the lateral ventricles, in the third ventricle, and the right suprasellar cistern, as well as in the bilateral eye globes (Fig. 1). The patient was suspected of having an intraventricular hemorrhage because the attending physician initially failed to associate the intraocular silicone oil with the intraventricular lesions. Further brain MRI in the supine position revealed abnormal signals in the bilateral eyeballs, bilateral lateral ventricles, third ventricle, and right suprasellar cistern, showing hyperintensity (relative to the cerebrospinal fluid), surrounded by low-signal chemical shift artifacts, on T_1_-weighted imaging (T_1_WI); variable signals on T_2_-weighted imaging (T_2_WI) (hypo- or hyperintensity); hypointensity on fluid-attenuated inversion recovery (FLAIR); no diffusion restriction (hypointensity) on diffusion-weighted imaging (DWI); and hypo- or hyperintensity on the corresponding apparent diffusion coefficient (ADC) sequence. The lesion in the anterior horn of the right ventricle was smaller on MRI than on CT, yet most of the lesions were unexpectedly found to have moved to the posterior horn of the right ventricle (Fig. 2). The final craniocervical CT angiography (CTA) showed multiple high-density foci on both eyes, in the bilateral anterior horns of the lateral ventricles (the lesion in the posterior horn had moved back to the anterior horn of the right lateral ventricle), in the third ventricle, and the right suprasellar cistern, without enhancement and the presence of an aneurysm (Fig. 3A and B), as shown by nonenhanced CT. However, the right optic nerve had a larger density than the left, which was close to intraocular silicone oil (Fig. 3C). A review of the head CT images obtained before intraocular silicone oil injection revealed no high-density intraventricular lesions, suggesting that the lesions on CT, MRI and CTA might have been silicone oil and that intraocular silicone oil had migrated into the ventricular system, optic chiasm, and suprasellar cistern. Because the patient had no symptoms of high intracranial pressure, no treatment was performed for intraventricular silicone oil migration. The patient was followed up by telephone until August 31, 2021 (5 years after intraocular silicone oil tamponade). Her vision was the same as before, and no headache, eye pain, nausea or vomiting were reported. Additionally, a further study with spinal MRI recently performed on this patient did not demonstrate silicone oil migration into the subarachnoid space of the spinal cord (Fig. 4).

## Discussion and conclusions

Migration of silicone oil from the vitreous body into the ventricles along the optic nerve is extremely rare. Silicone oil vesicles could be observed in the optic nerve sheath 24 h after silicone oil tamponade [34], in the optic nerve one month later [35], and in the lateral ventricle eight months later [3]. Even after removal, intraventricular silicone oil was observed in some cases during follow-up, indicating that silicone oil migration occurred before its removal [3, 11]. Retrobulbar migration of silicone oil vesicles may be related to the time of endotamponade and intraocular pressure. The epidemiology of silicone oil migration into the ventricles remains unclear because most patients have intraocular silicone oil removed 3–6 months after filling, and regular imaging during this period and beyond is not widely accepted. Some patients are incidentally found to have ventricular deposits of silicone oil, mainly because of delayed removal of silicone oil or failure to remove silicone oil. Among the 32 cases of intraventricular silicone oil migration summarized in this study, we found more women than men (17:15); the average age of these patients was 61 years (ranging from 15 to 87 years). Since intraventricular silicone oil migration is mostly found incidentally, the time interval between silicone oil endotamponade and intraventricular silicone oil observation remains unclear. According to this literature review, the time interval can be as short as 6.5 months and as long as 25 years. Most patients undergo intraocular filling with silicone oil for retinal detachment caused by diabetic retinopathy and are incidentally found to have intraventricular silicone oil due to the abovementioned reasons. In our case, bilateral retinal detachment was also caused by diabetic retinopathy and was treated with vitrectomy of both eyes and silicone oil injection.

The migration route of intraocular silicone oil into the ventricles remains controversial, especially regarding how the intraocular silicone oil enters the subarachnoid space around the optic nerve since the vitreous body of the eye is not anatomically connected to the subarachnoid space. Previous pathological studies have confirmed that silicone oil can migrate posteriorly through the interstitial space of the optic nerve. In the presence of high intraocular pressure, silicone oil can penetrate the cerebral pia mater in some locations to enter the subarachnoid space of the optic nerve [4, 36, 37] or can enter the subarachnoid space of the optic nerve directly through the atrophic optic disc [3]. Since the subarachnoid space of the optic nerve is connected to the intracranial subarachnoid space, after entering the intracranial subarachnoid space, silicone oil can enter the ventricular system through the fourth ventricular foramina (Luschka-Magendie foramina). Therefore, optic nerve and ventricular silicone oil deposits often coexist, just as silicone oil appeared in the optic chiasm and ventricle in our case. Additionally, the right optic nerve had a larger density than the left, which was close to the density of intraocular silicone oil, indicating that intraocular silicone oil migrated into the ventricles via the optic nerve. In some patients, postoperative intraocular pressure was reported to increase, and silicone oil tamponade was therefore assumed to potentially lead to an increase in intraocular pressure, which could, in turn, cause retrobulbar migration of silicone oil, with subsequent migration into the ventricles, in rare cases [2, 3, 6, 11]. In summary, the entry of intraocular silicone oil into the ventricular system may be related to congenital anatomic variations, optic nerve atrophy, and increased intraocular pressure.

Because of its high surface tension, silicone oil in the ventricle always appears as a uniform spherical high-density lesion on brain CT, and its CT value is often greater than 90 Hounsfield units (HU). The density of silicone oil is lower than that of the cerebrospinal fluid, and the position of the silicone oil in the ventricle is often variable. The silicone oil mostly floats at the top of the ventricle, and moves as the patients change position. Therefore, the prone examination position is often used to confirm the diagnosis [3, 4, 6, 10–14, 16, 26, 27, 29]. Similar features have not been observed in other intraventricular lesions, such as cerebral hemorrhages and brain tumors. By comparison, silicone oil varies more substantially on brain MRI. On T_1_WI, silicone oil mostly shows hyperintensity (relative to the cerebrospinal fluid); on T_2_WI, variable signals, including hypointensity, intermediate intensity, and hyperintensity, can be observed, and generally, no restricted diffusion is observed on DWI, as in our case. Because of the different precession frequencies of silicone oil and cerebrospinal fluid, intraventricular silicone oil exhibits unique chemical shift artifacts on T_1_WI and T_2_WI sequences, i.e., a high-signal curvilinear band on the edge of the lesion side and a low-signal curvilinear band on the edge contralateral to the lesion. Therefore, the imaging characteristics of silicone oil chemical shift artifacts and the movement of the silicone particles are important factors for diagnosing the intraventricular presence of silicone oil. Moreover, after injection of a contrast agent, silicone oil generally does not show enhancement on CT or MRI [3]. The silicone particles in this patient showed no enhancement on CTA either. In our case, although the patient was not in an ideal prone position during the cranial CT scan, we found that most of the silicone oil in the anterior horn of the right lateral ventricle had migrated into the posterior horn of the lateral ventricle when performing brain MRI on the patient in the supine position. Interestingly, the final CTA scan revealed that the silicone particles in the posterior horn had moved back to the anterior horn of the ipsilateral ventricle, suggesting that the silicone oil shifted its position as the patient changed position, which was captured in real time. This finding also suggests that the imaging results obtained for patients with intraventricular silicone oil reflect only a moment in the entire dynamic evolution process. At a recent follow-up, we performed spinal MRI on the patient but did not demonstrate a migration of silicone oil into the spinal subarachnoid space. This finding further suggests that silicone oil tends to float in the uppermost part of the ventricular system but has difficulty entering the subarachnoid space of the spinal cord.

Because the physical and chemical properties of the silicone oil used in surgical injection are extremely stable, silicone oil has good biocompatibility. In addition, the ventricle and the cistern system are rather spacious; thus, intraventricular migration of silicone oil may exert no effect on the human body. Most patients who experience intraventricular silicone oil migration have no clinical symptoms or only nonspecific clinical manifestations, such as headache, dizziness, or nausea. However, in rare cases, intraventricular silicone oil may block cerebrospinal fluid circulation and produce intracranial hypertension [11]. Notably, given the risk of retrobulbar migration of intraocular silicone oil, intraocular pressure and visual acuity should be regularly monitored, and cranial imaging examination should be carried out, if necessary. In the case of persistent high intraocular pressure, intraocular silicone oil should be removed as soon as possible.

In conclusion, this case report describes a dynamic process of silicone oil displacement in the supine position and provides a comprehensive imaging presentation. The moving pattern and a characteristic chemical shift artifact on MRI are key to the diagnosis and may help prevent unnecessary examinations or intervention.

## Data Availability

Not applicable.

## Abbreviations

MRI: Magnetic resonance imaging
CT: Computed tomography
T_1_WI: T_1_-weighted imaging
T_2_WI: T_2_-weighted imaging
FLAIR: Fluid-attenuated inversion recovery
DWI: Diffusion-weighted imaging
ADC: Apparent diffusion coefficient
CTA: Computed tomography angiography
HU: Hounsfield unit
